# *Plasmodium falciparum* Malaria Challenge by the Bite of Aseptic *Anopheles stephensi* Mosquitoes: Results of a Randomized Infectivity Trial

**DOI:** 10.1371/journal.pone.0013490

**Published:** 2010-10-21

**Authors:** Kirsten E. Lyke, Matthew Laurens, Matthew Adams, Peter F. Billingsley, Adam Richman, Mark Loyevsky, Sumana Chakravarty, Christopher V. Plowe, B. Kim Lee Sim, Robert Edelman, Stephen L. Hoffman

**Affiliations:** 1 Center for Vaccine Development, University of Maryland School of Medicine, Baltimore, Maryland, United States of America; 2 Howard Hughes Medical Institute, University of Maryland School of Medicine, Baltimore, Maryland, United States of America; 3 Sanaria, Inc., Rockville, Maryland, United States of America; 4 Protein Potential LLC, Rockville, Maryland, United States of America; BMSI-A*STAR, Singapore

## Abstract

**Background:**

Experimental infection of malaria-naïve volunteers by the bite of *Plasmodium falciparum*-infected mosquitoes is a preferred means to test the protective effect of malaria vaccines and drugs. The standard model relies on the bite of five infected mosquitoes to induce malaria. We examined the efficacy of malaria transmission using mosquitoes raised aseptically in compliance with current Good Manufacturing Practices (cGMPs).

**Methods and Findings:**

Eighteen adults aged 18–40 years were randomized to receive 1, 3 or 5 bites of *Anopheles stephensi* mosquitoes infected with the chloroquine-sensitive NF54 strain of *P. falciparum*.

Seventeen participants developed malaria; fourteen occurring on Day 11. The mean prepatent period was 10.9 days (9–12 days). The geometric mean parasitemia was 15.7 parasites/µL (range: 4–70) by microscopy. Polymerase chain reaction (PCR) detected parasites 3.1 (range: 0–4) days prior to microscopy. The geometric mean sporozoite load was 16,753 sporozoites per infected mosquito (range: 1,000–57,500). A 1-bite participant withdrew from the study on Day 13 post-challenge and was PCR and smear negative.

**Conclusions:**

The use of aseptic, cGMP-compliant *P. falciparum*-infected mosquitoes is safe, is associated with a precise prepatent period compared to the standard model and appears more efficient than the standard approach, as it led to infection in 100% (6/6) of volunteers exposed to three mosquito bites and 83% (5/6) of volunteers exposed to one mosquito bite.

**Trial Registration:**

ClinicalTrials.gov NCT00744133

## Introduction

Experimental infection of malaria-naïve volunteers by the bite of *Plasmodium falciparum*-infected mosquitoes has been a preferred means to test the protective effect of malaria vaccine and drug candidates in malaria-naïve volunteers. Experimental induction of malaria by the bite of infected mosquitoes has been reported in the literature for nearly 90 years [1], [2]. The conduct of challenge trials is complicated by the need for a mosquito insectary and expertise of personnel in rearing the insects, the transport of infected mosquitoes to the study site, and tight restrictions on mosquito-rearing and infection to coincide with vaccine or drug administration. If sporozoites could be manufactured, vialed, and transported to clinical trial centers around the world, and used to infect volunteers by needle and syringe inoculation, this would dramatically increase the capacity to assess new anti-malaria vaccine and drug candidates. Such capacity is of critical importance as the need for malaria challenge facilities is expected to grow with the advent of new malaria vaccine constructs and new antimalarial drugs [3].

Despite the current challenges to using infected mosquitoes for challenge, recent data from 532 volunteers has demonstrated that experimental infection of malaria-naïve volunteers is safe and reliable [4], and more than 1450 volunteers have been challenged by this method during the past 25 years [5]. The challenge model effectively predicted clinical efficacy for the RTS,S malaria vaccine prior to clinical trials in malaria-endemic areas [6].

Except in settings with very high malaria transmission intensity, individuals are rarely bitten by more than one infected mosquito per night under natural conditions. Malaria sporozoite challenge studies have traditionally relied upon the bites of five infected mosquitoes to induce malaria [7], [8], [9], [10]. Fewer bites by mosquitoes raised in non-sterile conditions, have not reliably induced malaria in volunteers [11], [12], [13], and increased bites may provide an unrealistic and overwhelming challenge in which even irradiated-sporozoite immunized volunteers fail to be protected from malaria challenge [14].

The aseptic rearing of *P. falciparum* sporozoite-infected mosquitoes in compliance with current Good Manufacturing Practices (cGMPs) and the demonstration that these mosquitoes contain fully infectious sporozoites that can transmit malaria infection is the first step toward developing a method to manufacture vialed sporozoites for parenteral administration. Furthermore, use of such mosquitoes to infect volunteers may reduce the variability of sporozoite load among mosquito lots, improve reproducibility of the pre-patent period and infection rate, and decrease the theoretical risk to human volunteers of infection by co-infecting microorganisms in the mosquito salivary glands. While laboratory-reared *Anopheles* typically have higher salivary gland sporozoite loads than wild-caught anophelines [15], they have highly variable loads [16], [17] which may result in a variable inoculum of sporozoites with each mosquito bite [17]. An increase in sporozoite and liver stage parasite burden may decrease the prepatent period [12], [18], and a decrease in burden could increase the prepatent period. In our experience *A. stephensi* mosquitoes reared in standard insectaries are contaminated with bacteria and fungi, which may reduce mosquito fitness and infection by the malaria parasite. There is no evidence from challenge studies that coincidental transmission of these agents to humans occurs but there is the theoretical risk of mechanical transmission of fungi and bacteria from the proboscis during feeding by traditionally-reared mosquitoes.

The production of aseptic mosquitoes, reared in compliance with cGMPs has been established for the manufacture of the metabolically active, non-replicating (radiation attenuated), aseptic, purified, vialed *P. falciparum* sporozoites (PfSPZ) used in the PfSPZ Vaccine [19], [20]. In this study we have used non-irradiated mosquitoes manufactured by the identical procedure as those used for the PfSPZ Vaccine to establish the infectivity of aseptic sporozoites transmitted by aseptically reared mosquitoes. We sought to determine the minimum number of *A. stephensi* bites required to safely achieve 100% volunteer infection with special attention to prepatent periods, sporozoite loads and parasitemia.

## Methods

### Objectives

The primary objective of this study was to evaluate the safety and tolerability of a new human malaria challenge model using aseptic *A. stephensi* mosquitoes infected with the chloroquine-sensitive NF54 isolate of *P. falciparum* and reared in compliance with cGMPs. Secondary objectives were to investigate the minimum number of *A. stephensi* bites required to safely achieve 100% volunteer infectivity, to study the character of the malaria infection and ascertain any differences between infection conferred by aseptic sporozoites and that described in traditional malaria challenge events, and to assess the role of molecular diagnostic techniques for accurate real-time diagnosis of *P. falciparum* infection. The protocol for this trial and supporting CONSORT checklist are available as supporting information; see Checklist S1 and Protocol S1.

### Study population and design

**Figure 1:**
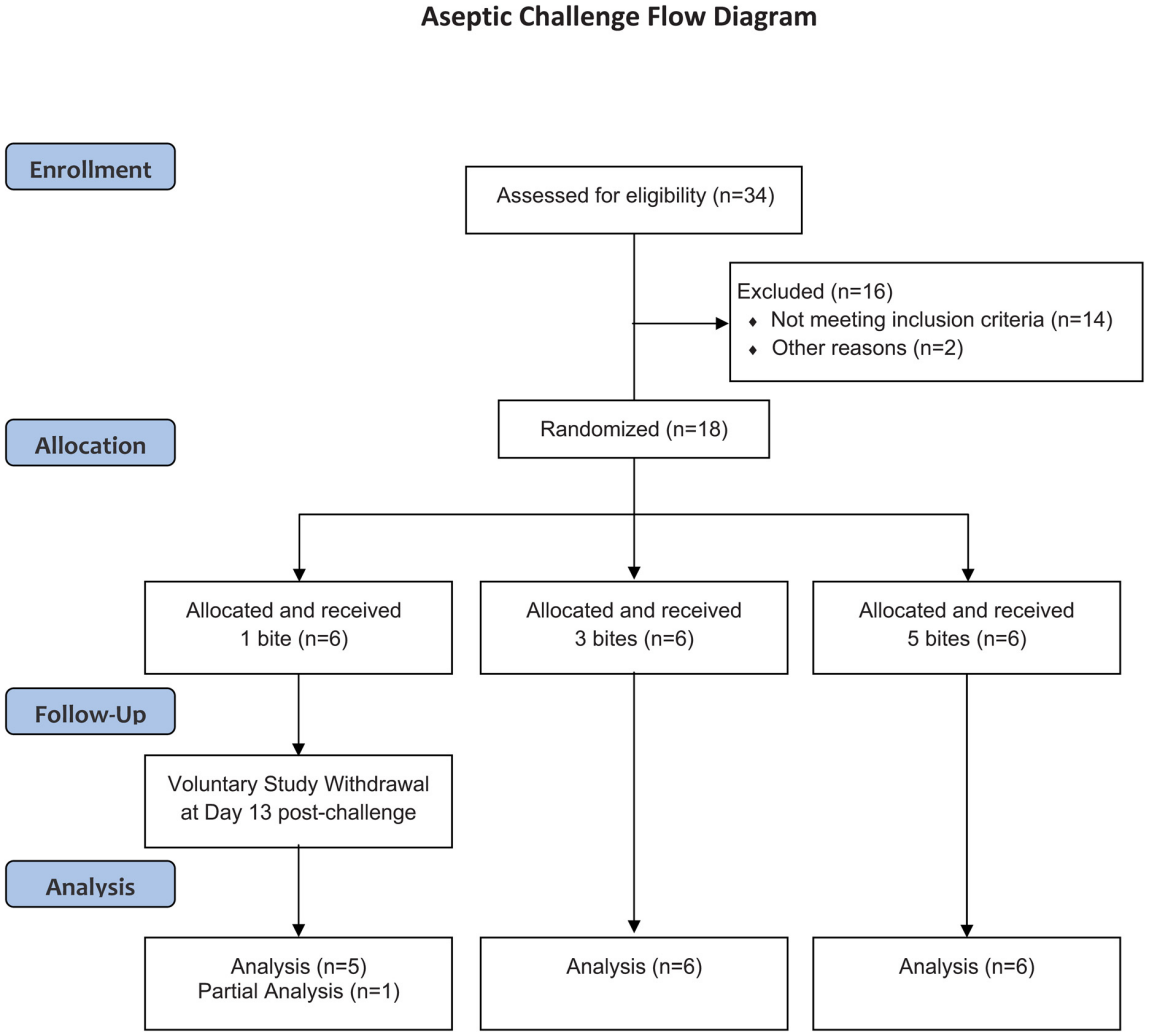
Study flow diagram.

The clinical study was conducted at the Center for Vaccine Development (CVD), at the University of Maryland School of Medicine in Baltimore, Maryland. Eighteen malaria-naive adults aged 19–39 years were randomized in a 1∶1∶1 ratio to receive 1, 3 or 5 bites of *A. stephensi* mosquitoes infected with *P. falciparum* (Figure 1). Participants were randomized by an online random allocation sequence generated by the EMMES Corporation and accessed by the study nurse coordinator. Once randomized, study personnel administering the challenge were not blinded as to the bite assignation. Participants were previously screened for good health and submitted blood for laboratories including hepatitis and HIV serologies and urine for pregnancy testing if applicable. Baseline complete blood counts (CBC), creatinine, glucose, aspartate aminotransferase (AST) and alanine aminotransferase (ALT) were screened and only volunteers with tightly defined normal values were enrolled. Additionally, volunteers were pre-screened for those with significant cardiovascular risk (i.e., >10%, 5 year risk)[21]. Risk factors include sex, age (years), systolic blood pressure (mm Hg), smoking status (current vs. past or never), body mass index (kg/mm^2^), reported diabetes status, or current treatment for raised blood pressure. A 12-lead ECG was performed and read by a staff cardiologist. Exclusion criteria included known history of malaria infection, long-term residence (>5 years) in a malaria-endemic area, travel to a malaria-endemic area within the previous 6 months, and splenectomy.

### Ethics

The trial was conducted in compliance with the Declaration of Helsinki. All volunteers signed an informed consent form after hearing a detailed explanation of the study and passing a written examination designed to ascertain if they understood the risks of malaria infection. Study protocols were reviewed and approved by institutional review boards of the University of Maryland and the National Institute of Allergy and Infectious Diseases/Division of Microbiology and Infectious Diseases. The trial was monitored by PPD, Inc. (Wilmington, NC).

### Dosage, Preparation and Administration of Study Product

*A. stephensi* mosquitoes infected with *P. falciparum* parasites of the NF54 strain were used for challenge utilizing methods developed by Sanaria, Inc (Rockville, MD). Briefly, eggs from *A. stephensi* mosquitoes were disinfected and placed in a custom medium for growth to pupae. Adult female mosquitoes were fed *P. falciparum* gametocytes in transfusion-quality human erythrocytes and serum. A proportion of the eggs, pupae, blood meal, and mosquitoes were cultured to assess for microbial growth. Only confirmed aseptic material was used in the study. Mosquitoes were transported under aseptic conditions to CVD and maintained aseptically at appropriate temperature and humidity. Prior to human challenge, one, three or five female mosquitoes were placed in a pint, cylindrical cardboard container with a mesh top. The mosquitoes were placed on exposed forearms and allowed to feed for 5 minutes, after which they were removed and dissected to determine whether the mosquito had 1) fed and 2) salivary gland sporozoites. A mosquito would be categorized as “fed” if blood was found within the mid-gut after challenge. Entomologist also dissected out the paired salivary glands and quantified the sporozoite load. If necessary, additional mosquitoes were used until the requisite numbers of infected mosquitoes had fed upon the participant. The techniques utilized were identical to those used as part of the development of the metabolically-active, replication deficient, whole-organism malaria vaccine (PfSPZ Vaccine).

### Quantification of salivary gland sporozoite load

By convention, the number of sporozoites present in mosquito salivary glands is categorized as: 0 (no sporozoites), 1 (1–10), 2 (11–100), 3 (101–1000), 4 (>1000) [17]. In this study, a hemocytometer was used to quantify the sporozoite load per mosquito (Chakravarty et al., manuscript in preparation). The method used had a lower limit of detection of 250 sporozoites per mosquito.

### Post-challenge Assessment

Participants were monitored for 30 minutes after challenge and asked to maintain symptom diaries until Day 7. They were evaluated on Days 5–7 and were admitted to an in-patient ward on Day 8 prior to the expected time of blood stage parasitemia. Daily histories, vital signs, physical exams, blood smears and real-time quantitative polymerase chain reaction (RTQ-PCR) assays were performed. Asymptomatic individuals were provided with pagers for rapid contact, allowed to leave the ward during the day, and return in the evenings for evaluation. Symptomatic or parasitemic individuals remained on the ward under clinical supervision. After confirmation of parasitemia by microscopy, volunteers received 1500 mg chloroquine base as standard first line therapy over 48 hours. The volunteers were discharged after documentation of three sequential negative blood smears and were followed weekly for 4 weeks followed by a final visit on Day 56.

### Assessment of safety and tolerability

All adverse events were graded for 1) severity (mild-easily tolerated, moderate-interfered with daily activity, or severe-prevented daily activity) and 2) relatedness (associated or not associated to the study product). Exceptions included fever (mild, >99.5–100.4°F; moderate, >100.4–102.2°F; and severe, >102.2°F), and erythema/induration (mild, 0–22 mm; moderate, 21–50 mm; and severe, >50 mm). The local challenge site was assessed and general solicited symptoms or signs evaluated. Local reactions that persisted beyond Day 2 were recorded as adverse events based on the reasoning that erythema, pruritis and induration are normal responses to mosquito bites during two days post- exposure. Solicited symptoms related to the malaria challenge (Days 2–7) (Table 2) and related to blood stage malaria began on Day 8 (Table 3) and continued for the duration of the volunteer follow-up or until a malaria diagnosis. Any other signs or symptoms were considered to be unsolicited.

Due to an adverse cardiac event that occurred in the setting of malaria challenge at another challenge center, ECGs and troponin levels were done on day 3 and 10 after the malaria diagnosis for exploratory purposes [22]. Of note, this cardiac event consisting of chest pain at rest in a young female volunteer occurred in the setting of malaria vaccine administration, subsequent active malaria infection and malaria eradication using Riamet®. The temporal association of the event with malaria challenge was likely circumstantial but the exact etiology of the chest pain remains unclear. Safety labs including a CBC, creatinine, glucose, AST and ALT were drawn on all days of a positive malaria smear, the duration of treatment and at each of four weekly follow-ups thereafter.

### Malaria Diagnostics

#### Blood smears

Beginning on Day 5, daily blood smears were performed to monitor for the presence of blood stage parasites. Blood smear intervals were decreased to every 8–12 hours if participants developed signs or symptoms consistent with malaria, until a diagnosis was established. Ten µL of blood were placed on a microscope slides in a 1×2 cm rectangle, heat-fixed and Giemsa-stained for *P. falciparum* parasites. Two trained investigators, blinded to randomization results, examined five separate passes along the 1 cm axis using the 100x oil immersion lens of calibrated microscopes. This was doubled to ten passes for symptomatic individuals. The five passes performed by two separate microscopists on different areas of the smear examined a total of 0.9–1.1 µL of blood. Parasites were quantified per µL. For positive smears, or if questions or discrepancies arose, a third trained investigator (MBL) was called on to read and quantify parasite burden. The minimum criterion for acceptance of a positive smear was identification of two unquestionable *P. falciparum* parasites confirmed by at least one investigator and MBL. All therapeutic decisions were based on the results of a positive blood smear.

#### Real-time quantitative DNA polymerase chain reaction (RTQ-PCR)

To evaluate and optimize methods for early molecular diagnosis of *P. falciparum* malaria in challenge trials, RTQ-PCR was performed on 0.5 mL of venous blood collected contemporaneously with blood smears using published methods with minor adaptation [23]. Standard curve cell counts were determined by FacsCaliber flow cytometer. PCR primers were based on the published sequence of the highly conserved [24], stage specific [25] *P. falciparum* 18S ribosomal RNA gene. Primer sequences were identical to the corresponding sequence of the NF54 strain. Samples were blinded and assays were run daily. Each sample was run in triplicate along with a water control. The data were analyzed using the Applied Biosystem 7300 Absolute Quantification Software. The assay sensitivity was determined to be 40 parasites/mL. Results were not utilized for therapeutic decisions.

### Statistics

The study was designed to be a proof-of-concept study and was not powered for statistically significant comparisons. Each subject's exposure to the randomized number of bites (1, 3, or 5) was considered a separate Bernoulli trial with ‘success’ defined as a positive malaria smear within 56 days of exposure. The agreement of RTQ-PCR to the blood smear analysis was conducted and presented using Pearson's Correlation as well as a linear regression analysis. These analyses were conducted on matched, paired smear and RTQ-PCR test results utilizing SAS (version 9.1.3, Cary, N.C.). The study database was managed by the EMMES Corporation (Rockville, MD).

## Results

### Study Population and Malaria Challenge Event

Eighteen adults aged 19–39 years (mean: 29 years) underwent challenge in March 2009 by the bite of 1, 3 or 5 bites of *P. falciparum-*infected mosquitoes on the same day (Table 1). Participants randomized to receive 1, 3 or 5 bites required a mean presentation of 2, 5.7 or 14.5 challenge mosquitoes, respectively. In total, 49% of female *Anopheles* mosquitoes presented for challenge ingested a blood meal and approximately 75% of the fed mosquitoes had detectable salivary gland sporozoites.

**Table 1:** Demographic information and study results by group and by malaria infectivity.

Target Bites (n)	Age (years)	Sex(%)	Body mass index	Total mosquitoes presented (mean per person)	Total mosquitoes taking blood meal^a^ (% of total presented)	Total Infected Mosquitoes^b^ (% of those taking a blood meal)	Geometric mean sporozoites c	Mean Pre- patent period in days	Geometric mean parasite density per µl	Mean day of initial PCR detection d
Infected (17)										
1 bite (5)	28.8	2F (40)	31.8	10 (2)	6 (60)	5 (83)	15,028	11.2	9.6	8.6
range	(19–39)		(24–45)	(1–3)			(3,000–41,000)	(11–12)	(4–21)	(7–11)
3 bite (6)	28.7	1F (17)	28.5	40 (6.7)	26 (65)	18 (69)	16,616	10.8	15.5	7.2
range	(20–34)		(21–49)	(5–10)			(2,500–57,500)	(10–11)	(8–32)	(7–8)
5 bite (6)	31.1	2F (33)	33.3	87 (14.5)	39 (45)	30 (77)	16,988	10.7	24.1	7
range	(23–39)		(28–46)	(9–27)			(1,000–56,000)	(9–11)	(8–70)	(7)
Uninfected (1)										
1 bite^e^ (1)	21	1F(100)	39	9	1 (11)	1 (100)	22,000	N/A	N/A	N/A
										
Total (18)	29	6F (33)	33	146	72 (49)	54 (75)	16,753	10.9	15.7	7.5
range	(19–39)		(21–49)				(1,000–57,500)	(9–12)	(4–70)	(7–11)

a Mosquitoes that were presented in challenge and subsequently had evidence of blood in the midgut as determined by on-site observation through a dissection microscope.

b Fed mosquitoes that had sporozoites in the salivary glands as determined by dissection after the challenge had been complete.

c The geometric mean was calculated only on fed, infected mosquitoes.

d The mean day of initial PCR detection refers to day post-challenge.

e This volunteer who did not become infected before treatment was unique in that the first 8 mosquitoes presented to the volunteer did not feed.

### Sporozoite load results

Mosquitoes were dissected immediately and the total sporozoite density determined. The geometric mean sporozoite load was 16,753 (range: 1,000 to 57,500) sporozoites per infected mosquito and did not vary appreciably between bite groups (Table 1). No infected mosquitoes with fewer than 1,000 sporozoites were found.

### Post-Challenge Safety Assessment

Solicited adverse events were collected on local reactogenicity to the challenge on Days 2–7 (Table 2). No severe adverse events were reported. Fifteen local reactogenicity events in ten volunteers were associated with the challenge. All symptoms were rated as mild and included erythema, induration and pain at the challenge site. There were 29 solicited adverse events with only one related to the malaria challenge event (malaise). Three mild events (malaise, myalgia, and arthralgia) and one moderate (arthralgia) event occurred on Day 6 in two volunteers from the 5 bite group, and were deemed associated with impending malaria infection and not to the challenge event itself. The remaining solicited events related to self-limited viral gastroenteritis experienced by several participants before the inpatient portion of the trial. Six unsolicited adverse events (pruritis) were reported during the post-challenge period, five of which were deemed mild and one moderate.

**Table 2:** Signs and solicited symptoms during the 7-day follow-up periods after challenge.

	Study Group											
	All Groups			1 Bite (N = 5)			3 Bites (N = 6)			5 Bites (N = 6)		
	None (%)	Mild (%)	Mod (%)	None (%)	Mild (%)	Mod (%)	None (%)	Mild (%)	Mod (%)	None (%)	Mild (%)	Mod (%)
Local												
Erythema	8 (44.4)	10 (55.6)	0 (0)	3 (50)	3 (50)	0 (0)	2(33.3)	4 (66.7)	0 (0)	3 (50)	3 (50)	0 (0)
Induration	14 (77.8)	4 (22.2)	0 (0)	5(83.3)	1(16.7)	0 (0)	4(66.7)	2 (33.3)	0 (0)	5 (83.3)	1 (16.7)	0 (0)
Site Pain	17 (94.4)	1 (5.6)	0 (0)	6 (100)	0 (0)	0 (0)	6 (100)	0 (0)	0 (0)	5 (83.3)	1 (16.7)	0 (0)
Systemic												
Malaise	10 (55.6)	5 (27.8)	3 (16.7)	3 (50)	2(33.3)	1 (16.7)	4(66.7)	1 (16.7)	1 (16.7)	3 (50)	2 (33.3)	1 (16.7)
Myalgia	15 (83.3)	1 (5.6)	2 (11.1)	5 (83.3)	0 (0)	1 (16.7)	6 (100)	0 (0)	0 (0)	4 (66.7)	1 (16.7)	1 (16.7)
Arthralgia	14 (77.8)	2(11.1)	2 (11.1)	5 (83.3)	1(16.7)	0 (0)	6 (100)	0 (0)	0 (0)	3 (50)	2 (33.3)	1 (16.7)
Nausea	13 (72.2)	2 (11.1)	3 (16.7)	4 (66.7)	0 (0)	2 (33.3)	5(83.3)	1 (16.7)	0 (0)	4 (66.7)	1 (16.7)	1 (16.7)
Abd. pain	16 (88.9)	2 (11.1)	0 (0)	5 (83.3)	1(16.7)	0 (0)	6 (100)	0(0)	0 (0)	5 (83.3)	1 (16.7)	0 (0)
Diarrhea	15 (83.3)	3 (16.7)	0 (0)	6 (100)	0 (0)	0 (0)	4(66.7)	2 (33.3)	0 (0)	5 (83.3)	1 (16.7)	0 (0)
Fever	18 (100)	0 (0)	0 (0)	6 (100)	0 (0)	0 (0)	6 (100)	0 (0)	0 (0)	6 (100)	0 (0)	0 (0)
Urticaria	18 (100)	0(0)	0 (0)	6 (100)	0 (0)	0 (0)	6 (100)	0 (0)	0 (0)	6 (100)	0 (0)	0 (0)
Headache	14 (77.7)	3 (16.7)	1 (5.6)	5 (83.3)	0 (0)	1 (16.7)	4(66.7)	2 (33.3)	0 (0)	5 (83.3)	1 (16.7)	0 (0)

### Malaria Event

Seventeen participants developed parasitemia, fourteen occurring on Day 11, with a mean pre-patent period of 10.9 days (9–12 days). The eighteenth participant (1 bite group) withdrew from the study on Day 13, and was PCR and smear negative on Day 13 at which point she was treated with chloroquine. Interestingly, it took exposure to 9 mosquitoes for one to successfully feed on this participant (Table 1). Six participants had mild symptoms including malaise, headache, nausea, and diarrhea preceding malaria diagnosis by a mean of 4.3 days (range 3–6). No individuals had temperatures greater than 99.9°F before detection of parasitemia. Most participants (15/18, 83.3%) reported at least one adverse event. Fifteen individuals developed an elevated temperature, five of which were graded as severe (>102.2°F) with a mean duration of 2.2 days. The most common solicited adverse events included fever (n = 15), headache (n = 13), malaise (n = 12), myalgias (n = 11) and chills/rigors (n = 11) (Table 3). All other solicited adverse events were graded as mild or moderate. Solicited symptoms peaked 48–72 hours after diagnosis coinciding with chloroquine clearance of parasitemia and persisted for a mean of 5 days (2–11 days).

**Table 3:** Signs and solicited symptoms during the malaria event by bite group.

			Study Group											
	Past Results^a^ (N = 47)	Current Results^b^ (N = 17)	1 Bite (n = 5)				3 Bites (n = 6)				5 Bites (n = 6)			
Symptoms	N (%)	N (%)	None N (%)	Mild N (%)	Mod N (%)	Severe N (%)	None N (%)	Mild N (%)	Mod N (%)	Severe N (%)	None N (%)	Mild N (%)	Mod N (%)	Severe N (%)
Fever^c^	47 (100)	15 (88)	0 (0)	3(60)	2(40)	0 (0)	1(17)	1(17)	2(33)	2 (33)	1 (17)	1 (17)	1 (17)	3 (50)
Headache	47 (100)	12 (71)	2(40)	1(20)	2(40)	0 (0)	1(17)	3(50)	2(33)	0 (0)	1 (17)	3 (50)	2 (33)	0 (0)
Malaise	44 (94)	12 (71)	3(60)	0 (0)	2(40)	0 (0)	1(17)	2(33)	3(50)	0 (0)	1 (17)	4 (67)	1 (17)	0 (0)
Chills	40 (85)	11 (65)	4(80)	1(20)	0 (0)	0 (0)	3 (50)	2(33)	2(33)	0 (0)	0 (0)	3 (50)	3 (50)	0 (0)
Myalgia	38 (81)	12 (71)	2(40)	0 (0)	3(60)	0 (0)	2 (33)	2(33)	2(33)	0 (0)	1 (17)	4 (67)	1 (17)	0 (0)
Nausea	29 (62)	6 (35)	0 (0)	2(40)	1(20)	0(0)	4 (67)	1(17)	1(17)	0 (0)	4 (67)	2 (33)	0 (0)	0 (0)
Dizziness	24 (51)	5 (29)	0 (0)	0 (0)	0 (0)	0 (0)	2 (33)	3(50)	1(17)	0 (0)	5 (83)	1 (17)	0 (0)	0 (0)
Arthralgia	17 (36)	10 (59)	2(40)	0 (0)	3(60)	0 (0)	2 (33)	0 (0)	3(50)	0 (0)	2 (33)	2 (33)	2 (33)	0 (0)
Abd. pain	17 (36)	5 (29)	3(60)	1(20)	1(20)	0 (0)	5(83)	0 (0)	1(17)	0 (0)	3 (50)	3 (50)	0 (0)	0 (0)
Diarrhea	12 (26)	5 (29)	4(80)	2(40)	0 (0)	0 (0)	4(67)	2(33)	0 (0)	0 (0)	5 (83)	1 (17)	0 (0)	0 (0)
Cough	9 (19)	0 (0)	0 (0)	0 (0)	0 (0)	0 (0)	0 (0)	0 (0)	0 (0)	0 (0)	0 (0)	0 (0)	0 (0)	0 (0)
Vomiting	6 (13)	3 (13)	4(80)	1(20)	0 (0)	0 (0)	6(100)	0 (0)	0 (0)	0 (0)	4 (67)	2 (33)	0 (0)	0 (0)
SOB^d^	ND	2 (12)	6(100)	0 (0)	0 (0)	0 (0)	5 (83)	1(17)	0 (0)	0 (0)	5 (83)	1 (17)	0 (0)	0 (0)
Stamina^d^	ND	3 (18)	4 (80)	1(20)	0 (0)	0(0)	4 (67)	2(33)	0 (0)	0(0)	6(100)	0 (0)	0 (0)	0 (0)
Chest pain	ND	0 (0)	0 (0)	0 (0)	0 (0)	0 (0)	0 (0)	0 (0)	0 (0)	0 (0)	0 (0)	0 (0)	0(0)	0 (0)
Urticaria	ND	0 (0)	0 (0)	0 (0)	0 (0)	0 (0)	0 (0)	0 (0)	0 (0)	0 (0)	0 (0)	0 (0)	0 (0)	0 (0)

a Previously published literature on the symptoms associated with the malaria event in the traditional malaria challenge[4].

b Summary data of findings noted at the University of Maryland at Baltimore.

c A fever was defined as >99.5°F orally.

d SOB refers to shortness of breath; stamina refers to change in exercise tolerance.

Laboratory abnormalities were noted in 12 of 17 (71%) individuals (Table 4). Abnormalities in liver transaminases (AST and ALT), platelets and white blood cell count were most commonly noted. One individual in the 3 bite group had severe, albeit clinically insignificant, thrombocytopenia (84,000/mm^3^) and three participants had mild or moderate thrombocytopenia. Five participants (27.8%) had abnormal AST or ALT levels with peak values of 202 IU/µL and 124 IU/µL. Six participants developed leukopenia. No hypoglycemic events or renal abnormalities were noted. All abnormalities resolved spontaneously after therapy. All ECGs and troponin levels were normal.

**Table 4:** Laboratory abnormalities recorded during the malaria event.

			Study Group		
Parameter/unit	Grade	Range	1 Bite (%)	3 Bites (%)	5 Bites (%)
AST (IU/L)	None	0–40	4 (80)	4 (67)	3 (50)
	Mild	41–99	1 (20)	2 (33)	1 (17)
	Moderate	100–199	0 (0)	0 (0)	2 (33)
	Severe	≥200	0 (0)	0 (0)	0 (0)
ALT (IU/L)	None	0–55 (♂) 0–40 (♀)^a^	5 (100)	4 (67)	3 (50)
	Mild	56–137 (♂) 41–99 (♀)	0 (0)	2 (33)	1 (17)
	Moderate	138–274 (♂) 100–199 (♀)	0 (0)	0 (0)	2 (33)
	Severe	≥275 (♂) ≥200 (♀)	0 (0)	0 (0)	0 (0)
Hemoglobin (g/dL)	None	12.5–17.0 (♂) 11.5–15.0 (♀)	5 (100)	5 (83)	6 (100)
	Mild	10.6–12.4 (♂) 11.1–11.4 (♀)	0 (0)	1 (17)	0 (0)
	Moderate	10,0–10.5 (♂) 9.6–10.0 (♀)	0 (0)	0 (0)	0 (0)
	Severe	<10.0 (♂) ≤9.5 (♀)	0 (0)	0 (0)	0 (0)
WBC (x 10^3^/mm^3^)	None	4.0–10.5	3 (60)	3 (50)	4 (67)
	Mild	2.5–3.9	2 (40)	2 (33)	2 (33)
	Moderate	1.5–2.4	0 (0)	1 (17)	0 (0)
	Severe	<1.5	0 (0)	0 (0)	0 (0)
Platelets (x 10^3^/mm^3^)	None	≥140	5 (100)	2 (33)	6 (100)
	Mild	125–139	0 (0)	0 (0)	0 (0)
	Moderate	100–124	0 (0)	3 (50)	0 (0)
	Severe	20–99	0 (0)	1 (17)	0 (0)

a (♂) represents males and (♀) represents females.

### Malaria diagnostics

**Figure 2:**
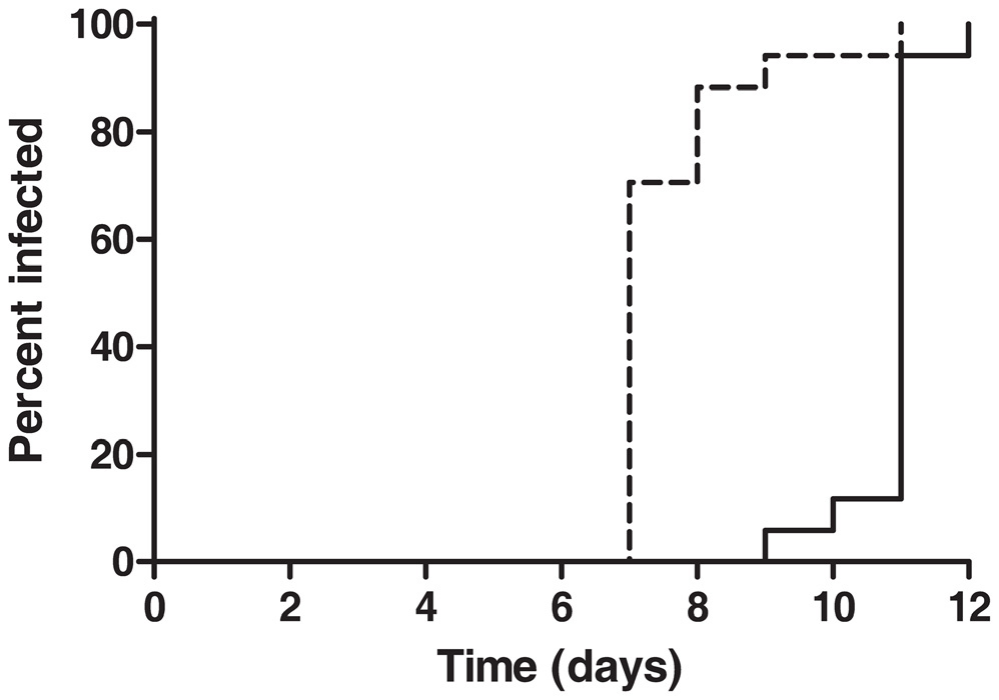
Time to infection of volunteers measured by blood smear and PCR. The time to infection of all volunteers (pooled exposed to 1, 3 and 5 infectious bites) measured by blood smear (solid line, median time to infection  = 11 days) and PCR (dashed line, median time to infection  = 8 days) was significantly different (p≤0.001, Mantel-Cox test).

**Figure 3:**
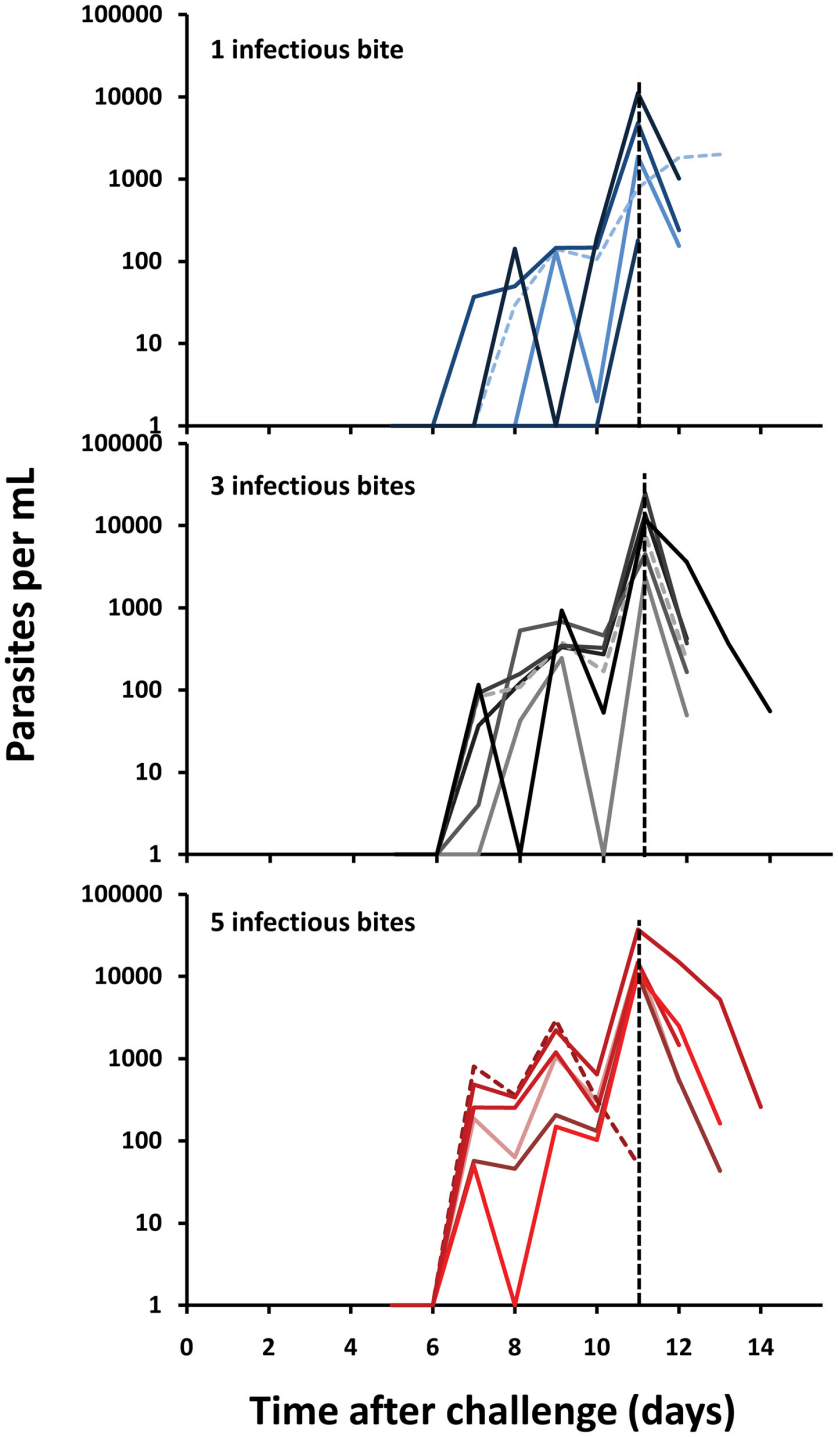
Dynamics of parasite growth in volunteers after challenge. Each line shows the parasite density in an individual volunteer as measured by PCR after being bitten by 1 (top), 3 (middle) or 5 (bottom) *Plasmodium falciparum*-infected *Anopheles stephensi* mosquitoes. All volunteers were treated on day 11 (vertical dashed line on each panel) when parasites were detected by blood smear, except for one volunteer in the 1 bite group (blue dashed line) who was positive and treated on day 12, one volunteer (grey dashed line) in the 3 bite group who was positive and treated on day 10, and one volunteer (red dashed line) in the 5 bite group who was positive and treated on day 9. Data are presented until last positive identification of parasites in the blood by PCR. Values shown as 1 on the log scale were negative.

The geometric mean parasite density at diagnosis as determined by blood smear analysis was 15.7 parasites/µL. A “dose response” was noted within the bite groups with the following geographic mean parasite densities for the 1, 3 and 5-bite groups (9.6, 15.5 and 24.1 parasites/µL) although the differences were not significant. While the mean pre-patent period was 10.9 days, the mean incubation period was 8.6 days. Detection by PCR was noted a mean of 3.1 days before positive smear results. There were no false positive or definitive false-negative PCR results although PCR failed to detect parasitemia in one individual (1 bite group) until the day of blood smear diagnosis. Removing this participant, PCR detection preceded blood smear diagnosis by 2–4 days (mean 3.3 days) (Figure 2). Furthermore, the dynamics of parasite growth by PCR, although limited by once daily monitoring, were quite similar for each bite group (Figure 3). Parasites were detected by blood smear for a mean of 1.94 days after initiation of chloroquine treatment and by PCR for a mean of 1.35 days after chloroquine initiation. There was a strong, statistically significant association between smear and PCR results (Pearson coefficient  = 0.841, 95% CI [0.763, 0.981], linear regression analysis R^2^ = 0.714, p = 0.002).

## Discussion

The use of *P. falciparum* sporozoite-infected *A. stephensi* mosquitoes reared aseptically under cGMPs is safe, efficiently and reliably transmits malaria, and is associated with a very precise pre-patent period. This first trial was a proof-of-principle study, establishing that these mosquitoes are viable and capable of transmitting malaria to volunteers at least as efficiently as mosquitoes reared under conventional conditions. Moreover, a precise prepatent period of 11 days was demonstrated in this study along with a determination that the quantity of mosquito bites required to confer malaria in 100% of the volunteers is at most, 3 bites, and may be one bite.

The traditional malaria challenge technique without the benefit of asepticity is safe and predictably transmits malaria [4]. The aseptic malaria challenge model appears similarly safe and well tolerated and advances the science of malaria challenge as well as the protection of human subjects. Symptom severity was generally mild to moderate with only five volunteers suffering a severe symptom (i.e. fever >102.2°F) defined by criteria consistent with FDA guidelines. The symptoms associated with malaria peaked 2–3 days after diagnosis coinciding with clearance of parasites, and thus probably represent an inflammatory response to the clearing infection. The solicited symptoms noted in this trial were generally mild and even less in frequency than previously reported (Table 4) [4]. Similarly, laboratory abnormalities were transient, and mild to moderate in grade with the exception of a single case of severe thrombocytopenia (84,000/mm^3^); a level that is not associated with an increased risk of bleeding. Leukopenia was noted in 41% of participants with malaria and occurred a mean of 2.1 days (1–4 days) after onset of detectable parasitemia by blood smear, and lasted between 1 and 8 days [4], [12], [26]. The severity of laboratory abnormalities did not appear to correlate with the bite quantity or sporozoite load within the biting mosquitoes. None of the symptoms or laboratory abnormalities met World Health Organization criteria for severe malaria [27].

The traditional challenge methodology relies on the bite of five mosquitoes to reliably transmit malaria to 100% of volunteers. Utilizing mosquitoes raised aseptically under cGMPs, all participants in the 3 and 5 bite groups and 5 of 6 volunteers in the 1 bite group developed malaria. One volunteer withdrew from the study on day 13 and was treated with chloroquine before developing parasitemia. Excluding this volunteer, whose ultimate infection status is unknown, 100% infectivity was achieved with a single bite. The number of mosquitoes required to achieve a successful blood feed varied per individual. In instances where a higher number of mosquitoes were required to achieve a successful blood feed, it is possible that the total number of mosquitoes to which the volunteers were exposed was underestimated as mosquitoes can inoculate sporozoites as they probe for blood, even if they did not take a blood meal. However, of the volunteers randomized to the one bite arm, two were exposed to the bite of only one mosquito and the rest were exposed to only two or three mosquitoes (total of 10 mosquitoes for infecting 5 volunteers, Table 1) before achieving a successful blood feed indicating that ≤3 mosquitoes are required to achieve malaria infection using this technique. It should be noted that all other studies have used the same criteria for a successful blood feed. Thus, regardless of probing without feeding, our data are distinct from the other studies, despite use of similar methodology.

Of the 17 participants who were infected, 14 (82%) developed a positive blood smear on Day 11 (range 9–12) after challenge. This compares favorably with previous published results where 18/47 (38%) participants developed malaria on Day 11 (range 9–14, mean 10.52 days) but the pre-patent period was more variable. Increased sporozoite inoculation and liver burden could result in reduced prepatent periods [18] or prolonged parasitemias [28]. Conversely, the physical characteristics of the mosquito salivary duct may limit the number of sporozoites that can be inoculated during probing and feeding [29]. In the *P. yoelli* model, sporozoite injection has proven to be highly variable ranging from 0–1,297 per bite (mean 123) and is only weakly correlated to sporozoite gland quantity [30]. Therefore, increased numbers of sporozoites per mosquito may not translate into increased sporozoites inoculated. Complicating interpretation, the traditional method of determining sporozoite loads is imprecise, relying upon qualitative estimation of total sporozoites on salivary glands squashes, with gland scores graded from 0 to 4+ [17]. In virtually all previous studies, mosquitoes with a gland score of ≥2+ (11–100 sporozoites) were considered infectious [7]. Utilizing a more precise counting technique, 16,753 sporozoites per mosquito (range 1,000–57,500) were present in the challenge mosquitoes. Our data indicate that mosquitoes raised aseptically in compliance with cGMPs can successfully transmit malaria to 100% of participants through the bite of 3, and likely 1, rather than 5 mosquito bites, and suggest they may lead to a more precise pre-patent period. The mosquitoes used in this study had more sporozoites than those used in most other studies. Thus, it is possible that the infectivity of 1 and 3 infected mosquitoes may have been due to an increased sporozoite inoculum when compared to traditionally-raised mosquitoes, but experimental data in *P. yoelii* [30] do not support this interpretation.

The use of PCR to achieve diagnosis earlier in malaria challenge studies is attractive, but it also carries risks. A false negative or false positive result in a small challenge trial of a malaria vaccine could profoundly alter study results. There were no false positive results in our preliminary use of this technique. While the ability to detect low-level parasitemia days before blood smear detection could, theoretically, avert symptoms associated with malaria, we did not find a correlation between PCR and symptom onset (data not shown). Moreover, the severity of symptoms increased once therapy was initiated and peaked after 48–72 hours when parasites were no longer detectable. Performance of the PCR assay could be increased to every 8–12 hours to enhance detection, but PCR is, currently, more labor-intensive procedure than is the reading of blood smears, and would require round-the-clock staffing in a challenge setting. Further study of diagnostic PCR in the context of volunteer challenges is required to fully document the pre-test specificity of the assay.

The development of a metabolically active, non-replicating (radiation attenuated) *P. falciparum* sporozoite vaccine is based on the successful immunization of volunteers by the bite of irradiated, non-aseptic mosquitoes [7]. It has been hypothesized that the contaminants, including bacterial and fungal material, accompanying these mosquitoes may provide some adjuvant effects that enhance sporozoite-induced immunity. Our study does not give any indication of the immunogenicity of the sporozoites administered by the bite of aseptically-reared mosquitoes, but establishing that the sporozoites produced under aseptic conditions remain fully virulent and capable of eliciting malaria is nonetheless important data for the effort to develop a metabolically active, non-replicating whole sporozoite *P. falciparum* vaccine, which is produced in aseptic *A. stephensi* mosquitoes using the same methodology.

Now that the aseptic malaria challenge model has been established with the NF54 strain of *P. falciparum*, which has historically been utilized in malaria challenge studies, we plan to develop challenges with additional *P. falciparum* strains. The genetic diversity of *P. falciparum* poses significant challenges to vaccine development [31], and the development of a heterologous challenge model will permit assessment of the ability of vaccine candidates to provide protection against genetically diverse parasites and of anti-malarial drugs to prevent malaria caused by *P. falciparum* of differing drug sensitivities.

The data from this trial demonstrate that aseptic sporozoites can transmit malaria. This is the first step toward developing and assessing the infectivity of aseptic, purified, cryopreserved *P. falciparum* sporozoites administered by needle and syringe to infect volunteers rather than relying on the bite of a mosquito. This would allow institutions without the capability of rearing infectious mosquitoes to safely, and reliably conduct malaria challenge trials, and such a study is being planned. Ultimately, our goal is to have multiple strains of *P. falciparum* parasites that are cryopreserved and packaged for transport to be used by institutions worldwide for testing malaria vaccines and pharmacologic agents.

## Supporting Information

Checklist S1 Consort Checklist
(0.23 MB DOC)

Protocol S1 Aseptic Challenge Protocol
(1.26 MB DOC)
